# Matrix therapy is a cost-effective solution to reduce amputation risk and improve quality of life: pilot and case studies

**DOI:** 10.1051/rmr/190002

**Published:** 2019-12-10

**Authors:** Pascal Desgranges, Taina Louissaint, Bertrand Godeau, Denis Barritault

**Affiliations:** 1 Department of Vascular Surgery, Hospital Henri Mondor, Université Paris-Est Créteil 94010 France; 2 Department of Endocrinology, Hospital Henri Mondor, Université Paris-Est Créteil 94010 France; 3 OTR3, SAS, 4 rue Française 75001 Paris France; 4 CRRET (EA 4397/ERL CNRS 9215), Université Paris-Est Créteil 94010 France

**Keywords:** Matrix therapy, CACIPLIQ20, OTR4120, ischemic skin ulcers

## Abstract

*Introduction*: Chronic, non-healing ulcers remain one of the most challenging clinical situations for health care practitioners. Often, conventional treatments fail and lead to amputation, further decreasing the patient's quality of life and resulting in enormous medical expenditures for healthcare systems. Here we evaluated the use of and cost-effectiveness of the RGTA (ReGeneraTing Agents) medical device CACIPLIQ20 (OTR4120) for chronic lower-extremity ulcers in patients with Leriche and Fontaine Stage IV peripheral arterial disease who were not eligible for revascularization. *Methods*: This uncontrolled pilot study included 14 chronic lower extremity ulcers in 12 patients in one hospital. The pilot study included 12 patients with TcPO_2_ < 20 mm Hg and ABPI < 0.5 who had either a minimum of one chronic lower extremity ulcer or a chronic ulcer related to amputation. OTR4120 was applied twice a week or until complete healing, for up to 12 weeks. Ulcer surface area reduction (%)after 2, 4, 8 and 12 weeks, appearance after 4 weeks, and healing after 12 weeks were measured and recorded. *Results*: A 35% reduction in ulcer size was achieved after 4 weeks. 7 (50%) out of 14 ulcers completely healed within 1 to 3 months of treatment. *Discussion*: OTR4120 is an effective therapeutic option for patients with chronic lower extremity ulcers, can provide major improvement of quality of life and has the added benefit of being a significant cost-effective solution for healthcare systems.

## 1 Introduction

The most favorable outcomes for patients with peripheral arterial disease (PAD) suffering from chronic, non-healing leg ulcers who do not promptly respond to standard wound care are achieved by restoring local blood flow through re-vascularization [15]. However, patients who do not qualify for re-vascularization are often left with no other option than amputation.

In the beginning of 2006, two patients from the provider's university hospital who were already scheduled for amputation were proposed, as a last resort, to be treated with the matrix therapy treatment CACIPLIQ20, also referred to as OTR4120, which was a biomedical research product at the time and was made exceptionally available for compassionate use. Within two weeks, these patients reacted so well that they were able to avoid amputation. A prospective study was rapidly submitted and accepted by the local ethical committee, who were aware that including a control group would be a great loss to the patients, as they would definitely be faced with amputation as their only treatment option.

OTR4120, the matrix therapy treatment kit used in this study, consists of in-situ applications of bioengineered structural analogues of heparan sulfate (HS) glycosaminoglycans, named RGTA (regenerating agents). At the site of a lesion HS are degraded, resulting in a disorganized extracellular matrix and destroyed tissue. RGTA replace damaged HS and provide a protective function by preventing the enzymatic degradation of communication peptides such as cytokines, chemokines and heparin-binding growth factors. They facilitate the processes of tissue repair and regeneration by restoring the extracellular matrix architecture and cell communication. RGTAs have been effectively used in preclinical models of the myocardium and acute limb ischemia as well as in models of pressure and necrotic skin ulcers [4,7,16,18].

The primary goal of this pilot study was to assess the utilization of a RGTA based medical device called OTR4120 to treat chronic lower-extremity ulcers in patients with peripheral arterial disease resulting from Leriche and Fontaine Stage who were not eligible for revascularization. Considering the enormous socio-economic impact of non-healing ulcers and lower-limb amputations, the authors have now re-visited this study retrospectively and have included a cost evaluation of OTR4120 treatment compared to conventional care as well as visual representation and details of the first two patients whose successful treatment led to this prospective study.

## 2 Materials and methods

This uncontrolled pilot study (assigned study number n°06-01) was authorized and approved by the hospital's institutional review board, the committee for the protection of people in biomedical research of Créteil-Henri Mondor Hospital (Le Comité Consultatif de Protection des Personnes dans la Recherche Biomédicale) on July 12th 2006 and was declared at the French agency for the safety of health products (l'Agence Française de Sécurité Sanitaire des Produits de Santé) on August 7th 2006.

### 2.1 Medical device and methods of use

The medical device used in this study is CACIPLIQ20^®^ (CE N°0499), also referred to as OTR4120, provided as a pack consisting of a 5 mL vial of RGTA OTR4120 (carboxymethyl glucose sulphate polymer) in sterile saline solution, a sterile gauze pad in a blister pack, and sterile forceps. Prior to the application of the OTR4120-soaked gauze pad, thorough mechanical debridement of the ulcer was performed, with a scalpel if necessary, in order to remove bacterial film, fibrin, exudates and dead tissue. The wound was then washed with ample volumes of sterile saline solution.

The application of the treatment is as follows: (1) without removing the gauze pad from the opened bister, the vial is entirely emptied onto the gauze pad, (2) using the forceps, the gauze pad is laid directly on the entire wound bed and then removed after 5 minutes of contact, (3) a non-adherent Vaseline bandage was used to cover the ulcer to protect the area during dressing changes, (4) this treatment was continued twice a week (every 3 to 4 days).

### 2.2 Patients

Patients were recruited at the Henri Mondor teaching hospital in Créteil, France over a 13 month period. Eligibility for the study included patients with Stage IV Peripheral Arterial Disease (Leriche and Fontaine Classification). Patients presented at least one chronic (present for at least 2 months without any signs of healing) lower extremity ulcer or a chronic ulcer linked with amputation. Additional requirements included values of ankle systolic pressure less than 70mmHg or toe systolic pressure less than 30 mmHg, arteriographic and Doppler evidence of severe peripheral arterial disease in the afflicted zone documented within the last 3 months. Moreover, ABPI (ankle brachial pressure index) values had to be below 50% and TcPO_2_ under 30mmHg. The only patients who were included were those not eligible for revascularization surgery. Non-inclusion criteria included: younger than 18 years of age, women with ineffective use of contraception, pregnancy, breastfeeding, eligibility for revascularization, recent (within 4 weeks) inclusion in another trial, social or psychological factors associated with an elevated risk of poor adherence, mental disorder which could inhibit the patient from showing or communicating pain, and incapability or objection to grant consent. Patients without health insurance were not included, as required by French law. All patients in this series provided written informed consent to have their cases and images published.

### 2.3 Baseline evaluation

Each patient's ulcer's characteristics, as well as age, sex, and co-morbidities were recorded. ABPI was calculated using measurements of systolic blood pressure taken at the brachial artery and ankle. When possible, TcPO_2_ was measured near the ulcer. Baseline characteristics for the patients are presented in Table 1.

**Table 1:** Characteristics of patients included in the study at baseline. ABPI, ankle-brachial pressure index; F, female; M, male; TcPO_2_, transcutaneous oxygen measurement.

Case #	Age (years), sex	Ulcer location	Ulcer duration (months)	Ulcer size at baseline (cm^2^)	Aspect at baseline	TcPO_2_ (mmHg)	ABPI
1	87M	Foot, medial aspect	12	0.5	100% F	NA	0.6
2	86F	Foot, medial edge	6	0.45	100% F	28	0.4
2	86F	Heel	6	1.05	30%F 70%G	28	0.4
3	85F	Left, medial aspect	10	9.1	10%F 90%G	7	NA
4	83M	Foot, sole	2	5.6	30%F 70%G	21	0.5
5	88M	Big toe, lateral aspect	>18	0.99	100%F	17	0.4
6	93F	Lateral malleolus	4	73.8	20%F 80%G	NA	NA
7	74M	Foot, lateral edge	>12	2.4	100%G	8	NA
8	63M	Heel	7	0.7	20%F 80%G	16	0.2
9	62M	Stump ulcer	1	49	20%F	27	NA
9	62M	2 weeks after femoral amputation		15	80%G	27	NA
10	89F		>12	0.6	50%F 50%G	69	0.4
11	78M		1	41.2		53	0.8
12	62M		2	24		NA	0.5

### 2.4 Treatment and time points

Application of the OTR4120-soaked gauze pad was twice a week (every 3–4 days) for 5 minutes until total healing, for up to 12 weeks. The gauze was removed and the wound was protected with a non-adherent, dry secondary dressing. Importantly, thorough debridement is a required step in order for OTR4120 to fully penetrate the wound bed and have its full effect. Treatment included applications of OTR4120 twice a week for 1 month.

A second month of treatment could be requested for those patients who had not totally healed by the end of the first month. A third month of treatment could be requested for those patients whose lesions persisted after 2 months.

Treatment effects were studied at day 3, weeks 1, 2, 3, and every 4 weeks until week 12.

### 2.5 Follow-up

Follow-up was possible since the majority of patients had to regularly return to treat their vascular disorders (Tab. 2).

**Table 2:** Characteristics of patients included in the study and wound evolution before and after CACIPLIQ20 treatment. M, male; F, female; F, fibrin; G, granulation tissue; SR, surface reduction; RF, right foot.

Case #	Age, years, sex	Ulcer location	Ulcer duration	Ulcer size at baseline (cm^2^)	Aspect at baseline	Appearance after 4 weeks	% SR after 2 weeks	% SR after 4 weeks	% SR after 8 weeks	Healing after 12 weeks	Commments	Follow up (months)	Alive at last follow up
1	87M	Foot, medial aspect	12	0.5	100%F	90%G 10%F	0%	0%	49%	Stopped	Left the study after 8 weeks, AHT	Lost to follow up	
2	86F	Foot, medial edge	6	0.45	100%F	20%F 80%G	44%	44%	80%	Healed	Hypertension, myocardial infarction, AHT	24	Yes
2	86F	Heel	6	1.05	30%F 70%G	Healed	100%	100%	100%	Healed			Yes
3	85F	Left, medial aspect	10	9.1	10%F 90%G	90%G 10%F	0%	0%	23%	Stopped	Bacterial infection between 4 and 8 weeks HTA healed later	24	Yes
4	83M	Foot, sole	2	5.6	30%F 70%G	100%G	4%	36%	49%	Not healed	Renal dialysis, died months after study completion from unrelated causes, HTA	16	Yes
5	88M	Big toe, lateral aspect	>18	0.99	100%F	70%F 30%G	19%	0%	0%	Not healed	Died from cachexia, HTA, smoker	2	No
6	93F	Lateral malleolus	4	73.8	20%F 80%G	95%G	13%	13%	27%	Healed other slowly healing ulcers, HTA	Lost to follow up		
7	74M	Foot, lateral edge	>12	2.4	100%G	100%G	37%	62%	100%	Healed		24	Yes
8	63M	Heel	7	0.7	20%F 80%G	100%G	14%	29%	−50%	Not healed	Bacterial infection, bladder carcinoma, HTA	24	Yes
9	62M	Stump ulcer	1	49	20%F	100%G	11%	46%	100%	Healed	Diabetes	12	Yes
9	62M	2 weeks after femoral amputation		15	80%G		0%	78%	100%	Healed		12	Yes
10	89F		>12	0.6	50%F 50%G	100%G	0%	40%	100%	Healed	HTA	Lost to follow up	
11	78M		1	41.2		90%G	0%	24%	56%	Not healed		24	Yes
12	62M		2	24		30% SR stopped the treatment	20%	13%	NA	Not healed	Amputation of 4 toes 3 months later then healed with CACIPLIQ20^®^ (outof the study), HTA, diabetes, smoker	24	Yes
Total								1 healed, remain 13	5 healed remain 12	7 healed remain 7		8 alive	

### 2.6 Endpoints

The percentage of ulcer healing was the primary endpoint. This was computed by dividing the ulcer surface area at week 4 by the surface area at baseline. Ulcer surface area was determined by multiplying the longest ulcer diameter by the shortest ulcer diameter; measurements were made using a graduated disposable ruler.

Secondary endpoints included (i) the percentage of ulcer healing (same assessment as above) at: week 1, week 2, week 3, week 8, and week 12; (ii) analgesic use determined by patient interviews at weeks 4 and 8; and (iii) requirement for amputation. Analgesic classification taken from the WHO pain relief ladder.

### 2.7 Statistical analysis

Changes in ulcer surface area between treatments were determined using the paired Student *t*-test (*P* values below 0.05 were considered significant).

### 2.8 Cost effectiveness

To assess the costs related to wound management using OTR4120 versus conventional care, data was sourced from the French National Health Care Annual Report of 2014, which calculated the associated costs of chronic wound care and then compared it with the cost of OTR4120 treatment [13]. Detailed reports from the French national agency ATIH's (Agence Technique de l'information sur l'hospitalisation) healthcare expenditures specific to amputations were also used in order to assess related costs. Published literature was also searched for reported data of the prevalence and cost of lower limb amputations to consider in this evaluation.

## 3 Results

### 3.1 First two patients whose results led to the pilot study

**Fig. 1:**
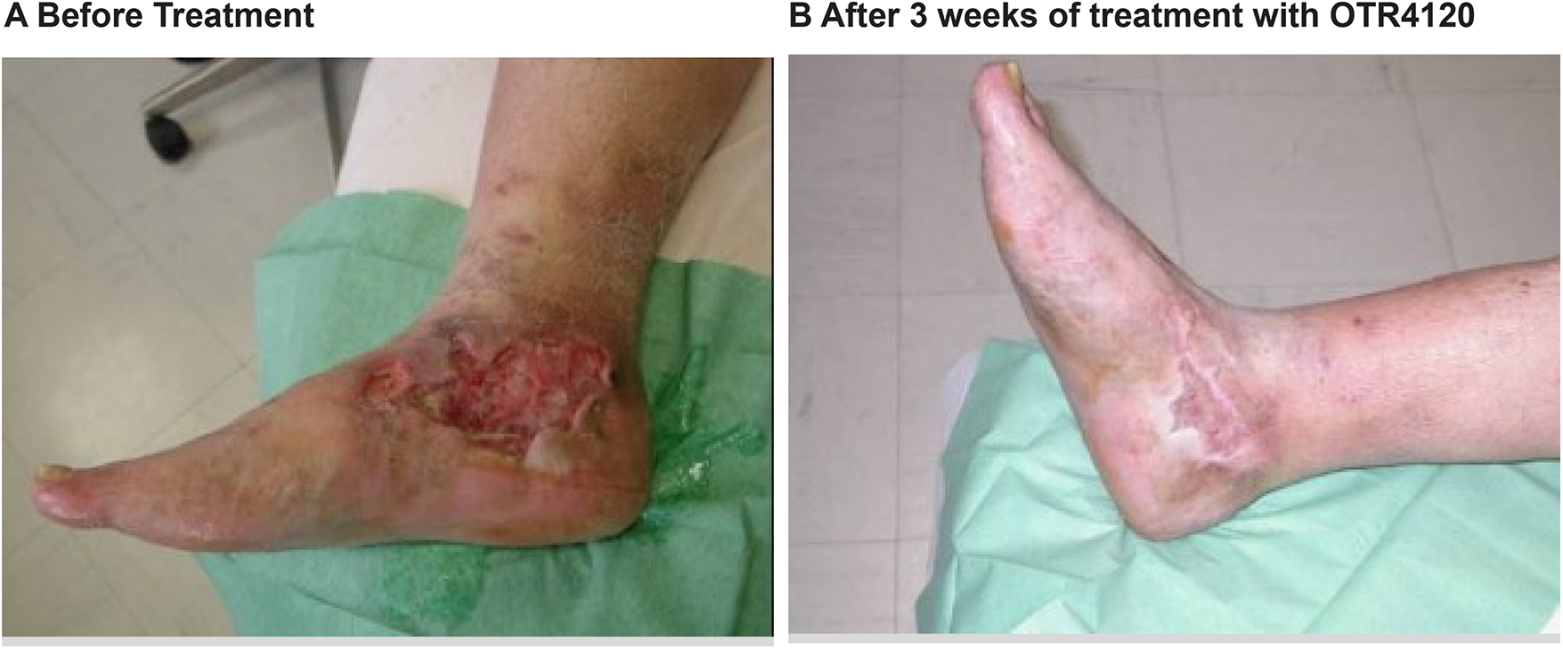
Case 1 (a) 77 year old patient with an arterial venous ulcer that had not shown improvement for 7 months before treatment was scheduled for amputation. (b) After 3 weeks of treatment with OTR4120, the wound size had been greatly reduced and showed no signs of inflammation. Amputation was avoided and the patient no longer required painkillers.

The following two cases were the first patients who were treated as a last resort with OTR4120 in the investigators' hospital unit and whose remarkable healing led to the approval of a pilot study by the ethical committee. The first patient was a 77 year old man with an arterial venous ulcer that had not improved for the last 7 months (Fig. 1A). Despite taking a maximal oral dose of opioids, this patient suffered around the clock from intolerable, excruciating pain. In desperation, the patient requested amputation with hopes to relieve the pain and was scheduled to be amputated a week before he was proposed the option of compassionate care treatment with OTR4120 as a last chance before amputation. The patient felt pain relief following the treatment and within a few days decided to continue with OTR4120 and canceled his amputation. Within 2 weeks his pain had disappeared and opioids were no longer necessary. At 3 weeks, the patient's wound had been greatly reduced and no longer showed signs of inflammation (Fig. 1B). The wound completely healed and the patient died a few years later from other causes, with both of his legs.

**Fig. 2:**
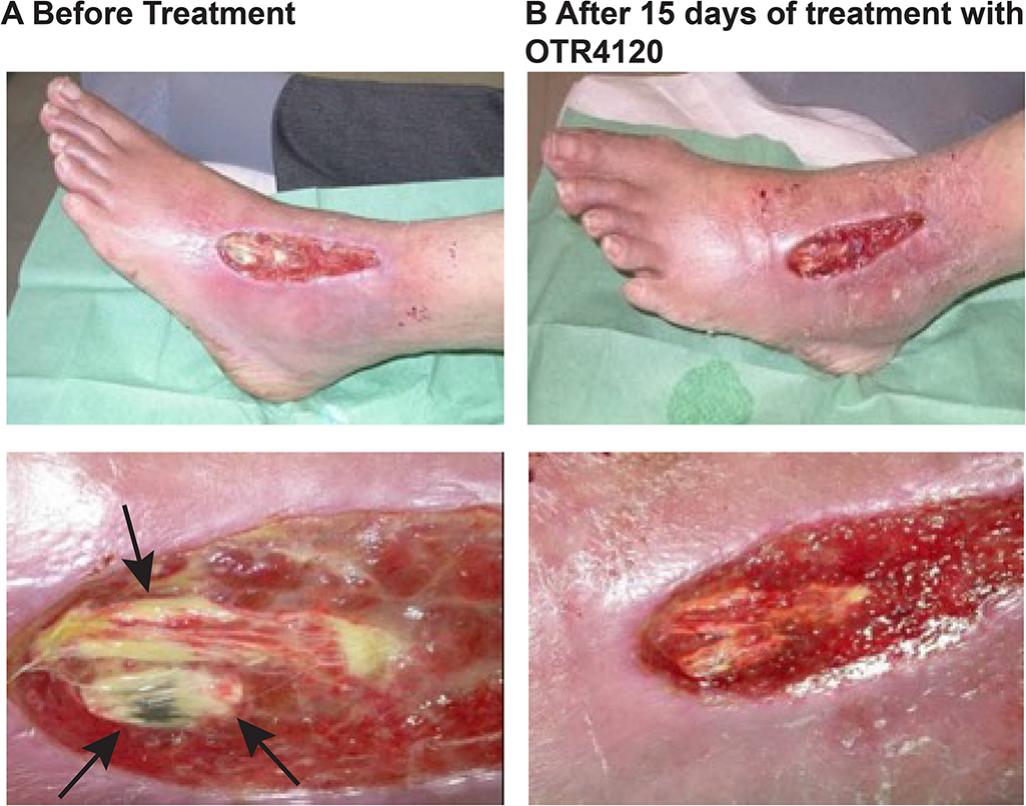
Case 2 (a) 65 year old patient with an ischemic, diabetic, neuropathic foot ulcer that had not shown improvement for 6 months before treatment, presenting with a necrotic lesion (black arrows) on the ligament and no granulation tissue (b) After 2 weeks of treatment with OTR4120, the necrotic zone diminished and granulation tissue formed, allowing the patient to avoid amputation and to be eligible for a skin graft.

The second patient was a 65 year old man with an ischemic and diabetic (and neuropathic) ulcer presenting necrotic tissue which increased in size daily, despite intensive conventional care, which aimed to prevent further development of necrosis (Fig. 2A). Amputation was expected in the upcoming weeks due to the high risk of gangrene as a result of ensuing necrosis. OTR4120 was proposed as a last chance to prevent this outcome. Within a few days, the size of necrotic tissue was reduced and in 2 weeks granulation tissue had covered most of the wound, allowing the option for a skin graft to help accelerate wound closure. (Fig. 2B).

### 3.2 Pilot study

15 patients were included in this study, including three who had well-controlled diabetes. Three patients were excluded from the analysis: One patient who died after two weeks from metastatic cancer, one patient who requested an amputation after the first week, and one patient who quit the study after the first week. Two patients presented two ulcers each, which amounted to a total of 14 ulcers for the study. The patient who had two ulcers simultaneously had them at two different sites. Thus, these ulcers were counted as two independent ulcers.

At baseline, average ulcer surface area was 14.15 cm^2^ (ranging from 0.5 to 73 cm^2^), with a 7.5 month average ulcer duration (1–18 months) (Tab. 1). Ulcer locations and appearance are also reported in Table 1. Two patients (#9 and #11) had amputation stump ulcers; they had started the treatment less than one month since amputation.

Ischemia was assessed after angiography and Doppler, TcPO_2_, and ABPI values. Table 1 includes the TcPO_2_, and ABPI results for each patient. Two patients had TcPO_2_ higher than 30% or ABPI exceeding 0.5, but met all other recruitment conditions and were not candidates for vascular surgery.

### 3.3 Safety

No adverse effects of OTR4120 were recorded. OTR4120 was well-tolerated.

### 3.4 Ulcer surface area

**Fig. 3:**
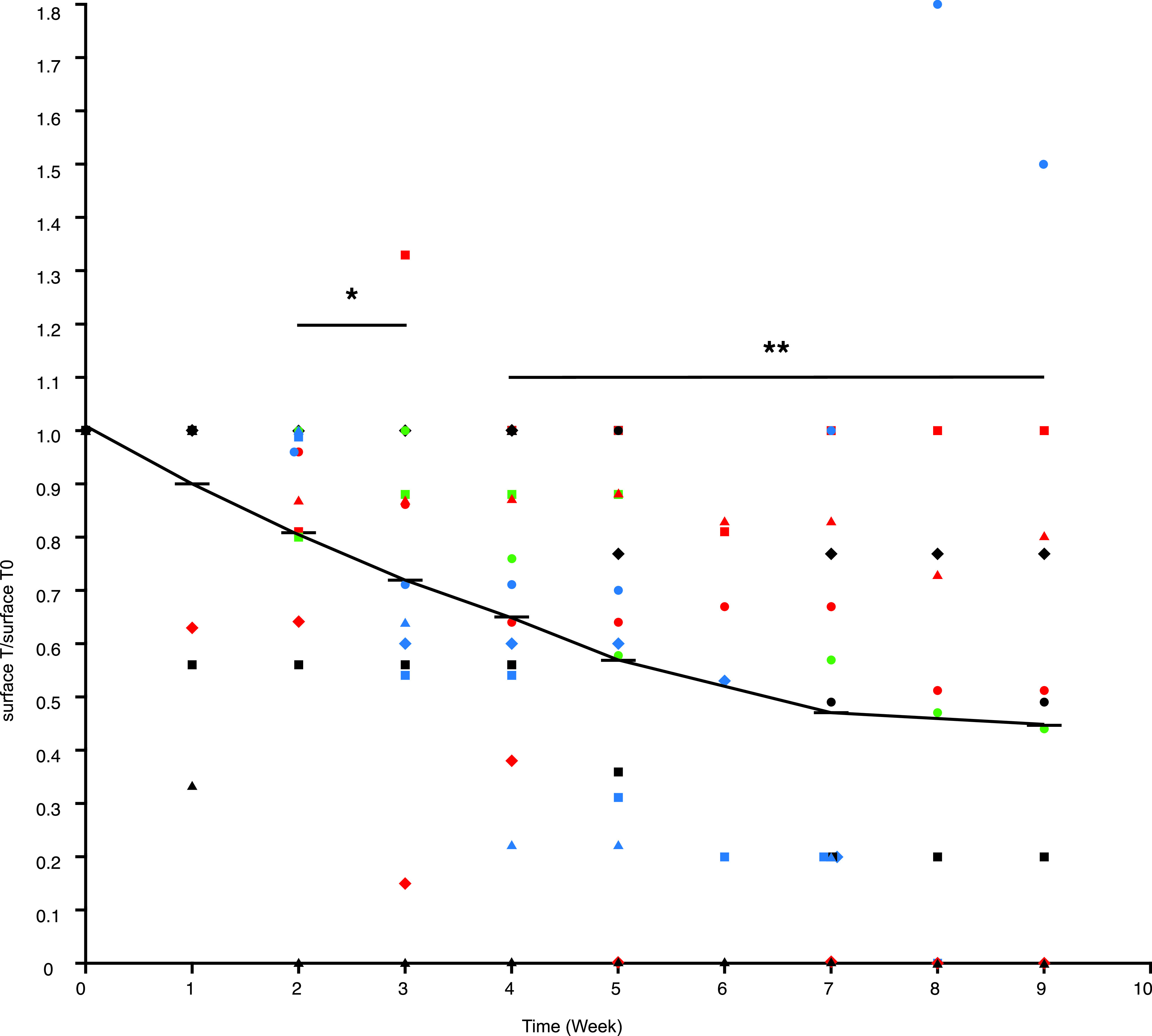
Ulcer size reduction during OTR4120 treatment. Changes in ulcer size during OTR4120 treatment per patient and treatment week. Mean ulcer surface area at each time point was divided by ulcer surface area at baseline and is shown for each patient and treatment week. The horizontal bars represent the average for each time point. *P ≤ 0.05, ** *p* ≤ 0.01.

As seen in Figure 3, the mean ulcer area for all patients decreased over the course of OTR4120 treatment. Ulcer surface area reduction was significant as soon as week 2 versus baseline (primary endpoint; 19%, *p* < 0.02) and even more significant at 4 weeks versus baseline (35%, *p* < 0.001; Fig. 3). During treatment with OTR4120 the surface area with fibrin was routinely decreased while the surface area with granulation tissue increased (Tab. 2). Table 2 presents ulcer surface reduction and healing status after 2, 4, 8 and 12 weeks of treatment with OTR4120. Of the 14 ulcers, 9 showed evidence of healing and one ulcer totally healed (complete closure) at 2 weeks. At 4 weeks, 7/12 cases showed reduction from 2 weeks of at least 15% and up to 43 %. At 8 weeks, 9/12 cases showed ≥50% surface reduction of ulcer size (Tab. 2). All of the patients with un-healed ulcers at 4 weeks decided to continue treatment. Four more ulcers were closed by 8 weeks (Tab. 2). Two patients (#5 and #8) did not show any improvement); one of them died 2 months later from cachexia syndrome and the second had bladder cancer. Six patients (6 ulcers) of the 8 patients (8 ulcers) who had unhealed ulcers at week 8 requested a third month of treatment. Two more ulcers healed (complete closure) at week 12. In the end, 50% of the ulcers had healed at 3 months (7 of 14 ulcers; Tab. 2).

### 3.5 Follow up (Tab. 2)

Of the initial 12 patients, 9 were followed for two years. At 12 months, 8 patients were still alive and 6 of them at 24 months. None of the closed ulcers re-opened and none of the patients were amputated. One of the 6 patients whose ulcer had not healed by 23 weeks eventually experienced wound closure and was still alive 2 years later. Three patients' ulcers did not change in size or appearance after the end of the treatment. None of the patient's ulcers treated with OTR4120 needed amputation . One patient's leg was amputated, (the other leg, not the one treated with OTR4120). Two patients died from causes unrelated to the ulcer (Tab. 2).

### 3.6 Pain and analgesic consumption

**Fig. 4:**
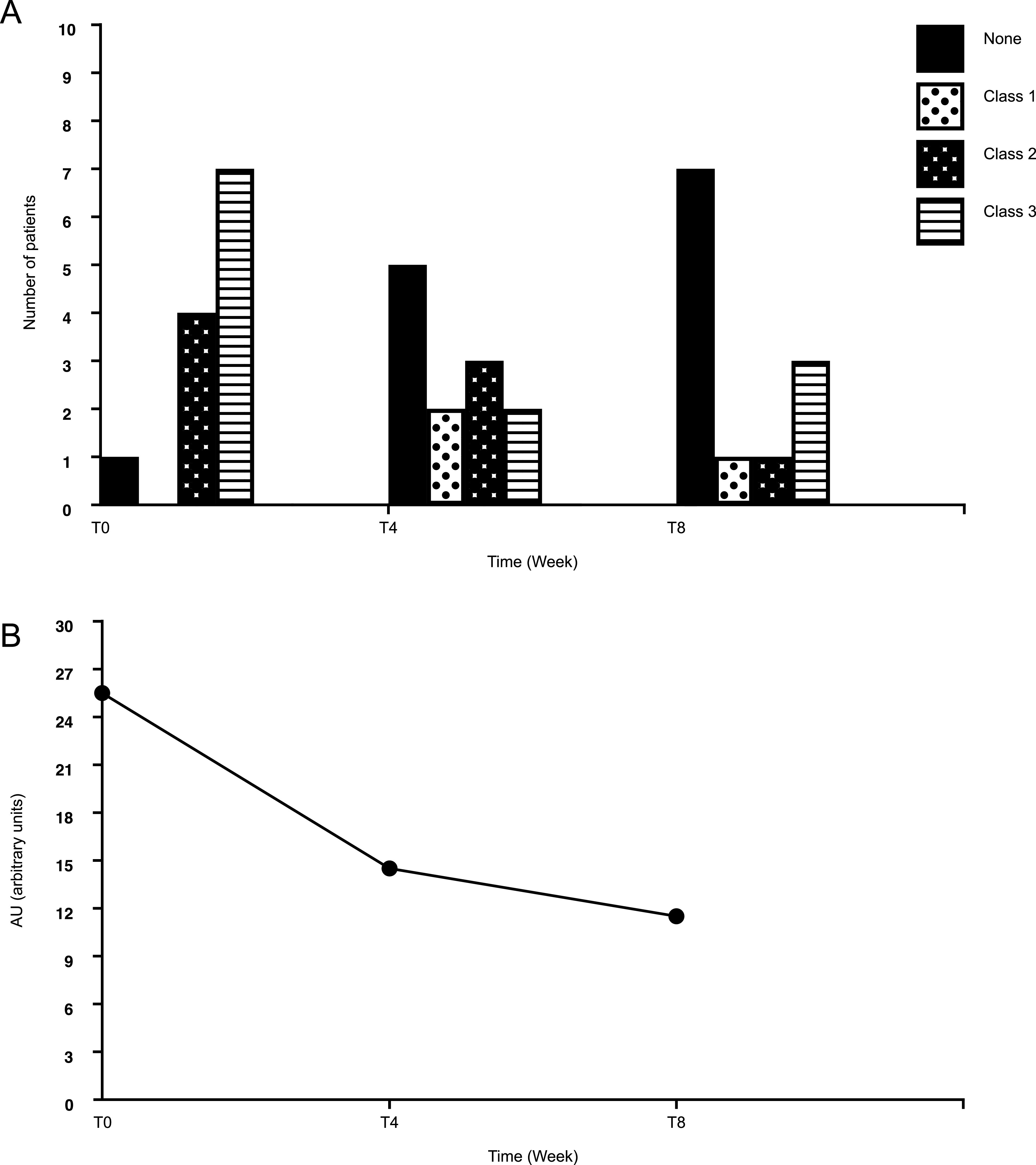
Evolution of analgesic consumption over the treatment period. (a) Evolution of the analgesic class over the treatment period. The majority of patients at T0 were taking class 3 (striped bars) analgesics at T0, which decreased over the course of the treatment. (b) Overall analgesic consumption decreased over time, calculated by the sum of the patients of the analgesic class multiplied by the corresponding arbitrary unit (none = 0 AU, class 1 = 1 AU, class 2 = 2AU, class 3 = 3AU). AU, arbitrary unit.

Analgesic use is shown in Figure 4. 1 arbitrary unit (AU) was assigned to step 1 analgesics, 2 AU were assigned to step 2 analgesics, and 3 AU were assigned to to step 3 analgesics. Eleven patients were using analgesics at baseline. These included 4 who were taking class 3 analgesics and 7 who were taking class 2 analgesics (Fig. 4A).The AU number decreased from 27 at the beginning of the treatment to 14 at 4 weeks (51.8%) and 11 (60%) at 8 weeks (Fig. 4B).

### 3.7 Cost effectiveness

Theoretically, accelerated healing time achieved with OTR4120 has the potential to save the health care system a significant amount per beneficiary. However, this amount would greatly depend on the health care coverage of OTR4120 in each country, as OTR4120 treatment may cost as much as 200 Euros per week of treatment. One may argue that the short-term cost of treatment may be higher. However, the long-term savings are enormous and undoubtedly outweigh the short-term cost. Indeed, if we consider that 50% of patients with non-healing critical ischemia chronic ulcers require amputation, treatment with OTR4120 could lead to massive savings for the health care system since none of the patients in this report required amputation in follow-up. Each year about 150,000 lower extremity amputations secondary to peripheral vascular disease (PVD) or diabetes are performed in the US alone, with a cost of about 60,000 dollars (in 2010) per patient, not including follow-up care [8,9].

Thus, treatment with OTR4120 could potentially prevent 75,000 amputations and save the health care system at least 9 billion dollars per year. Since this study was conducted in France, the authors consulted the French national agency ATIH (Agence Technique de l'information sur l'hospitalisation) for detailed healthcare expenditure reports. In France, in 2017, a total of about 11,000 amputations were performed for patients with peripheral arterial disease, with an average hospital stay of about 11 days and hospitalization for amputations that costed on average 14,000 euros per day [3]. Thus, by avoiding amputations, the French healthcare system could save at least 1.7 billion euros per year. In this report, we were able to prevent amputation for all 12 of our patients, which in present-day would have amounted to about 1.8 million euros of savings.

## 4 Discussion

The patients in this study who had chronic lower-extremity ulcers due to limb ischemia and were not eligible for revascularization had no other option than amputation. None of them were expected to show any signs of healing, since they had not responded to conventional therapy before the study. Here, the investigators report a matrix therapy effective in treating chronic non-healing ulcers, with 35% of ulcers healed at 4 weeks versus baseline. Moreover, 50% (7 out of 14 ulcers) achieved complete healing within 1 to 3 months of treatment with OTR4120. Among all of the treated ulcers, all showed signs of healing except for 1 patient who died shortly after the study ended. OTR4120 resulted in healing despite low TcPO_2_ and ABPI values. Ulcers that had not shown any signs of healing for several months started to form granulation tissue rapidly upon treatment application. Reduction of ulcer size was significant as early as two weeks and increased at 4 weeks until the end of treatment.

### 4.1 Tolerance and pain relief

Although in theory, OTR4120 could generate local skin type IV delayed hypersensitivity with redness, swelling and rush as induced by all heparin-like agents, these side effects were not observed in this study, as all patients tolerated the treatment very well [5].

In addition to improved healing, patients in this study also experienced striking pain relief. The pain relief effect of OTR4120 is an added advantage and quite valuable when considering the beneficial impact on quality of life. This effect has also been experienced by patients treated with RGTA matrix therapy for corneal ulcers, whose VAS pain score decreased from 72/100 at baseline to 50/100 after one week and down to 29/100 after four weeks of treatment (*p* < 0.001) [6]. Interestingly, the use of heparin for pain relief has been well noted and has been shown to relieve pain in burn patients [14]. It is well known that pain reduction is associated with healing. In the case of RGTA it is an unexpected consequence and indirect proof of its healing capacities.

Regarding the use of analgesic consumption, it is worth highlighting that not only did the overall amount of analgesic use decrease over the course of our study, with the majority of patients no longer requiring any by the end (Fig. 4B), but additionally, the profile of analgesic classes also improved: 7 patients were taking class 3 analgesics and 4 were taking class 2 at the beginning of the study, while 3 were taking class 3 and 1 was taking class 2, with the remaining 7 patients taking none (Fig. 4A).

### 4.2 Present-day format, use and cost-effectiveness of OTR4120

Since this study was completed, a spray form of the same OTR4120 solution was developed, making the product 10 times more economical and user-friendly (10 week treatment costs 200 euros). Indeed, three to four presses of the spray (each press delivers 140 μL of the OTR4120 solution) were enough to saturate 40 cm^2^ of a wound.

As 80% of amputations are caused by non-healing ulcers, it appears obvious that enhancing wound healing could reduce amputation rate and health care expenditures. In addition to decreasing the enormous economic burden, the possibility of faster complete wound closure, especially in patients with no other therapeutic option than amputation, also relieves the physical and emotional burden of non-healing recalcitrant wounds, consequently improving the patient's prognosis and quality of life. An advanced wound care product's cost-effectiveness is steered by its efficacy of accelerated and complete wound healing, which in turn reduces rates of complications, hospitalization, and amputation [11]. Indeed, although the use of advanced wound care products such as OTR4120 may increase short-term expenditures, cost can be greatly decreased over the long-term through increased healing rates, faster time-to-heal and decreased incidence of infection and amputation [2,19].

It is important to take note that recommendations from the manufacturer for using OTR4120must be followed carefully. A crucial step is to perform thorough, proper debridement of the wound. Notably, removing fibrin is crucial since fibrinogen can bind heparin through multiple binding sites and could bind OTR4120.

### 4.3 Limitations of the pilot study

The two limitations of our study include the limited number of enrolled patients (12), which makes the lack of adverse effects recorded difficult to affirm, and the lack of control patients who did not receive OTR4120 treatment. Indeed, the fact that the patients in this study did not have any other treatment option other than amputation renders finding control patients who would essentially agree to not receive any treatment quite challenging. Furthermore, the unexpected and successful healing of the first two patients treated by compassion with OTR4120 had made the inclusion of a controlled group difficult to defend or justify according to the directive on the use of placebos in the declaration of Helsinki [10,17].

## 5 Conclusions

Considering the poor prognosis of the 12 patients in this prospective pilot study, it would have been expected for half to be either deceased or amputated as a result of the ulcer during the course of the study that year. Astoundingly, none of them required amputation and even two years later, none were amputated or deceased as a result of the ulcer. After this study ended, 30 more patients in the provider's hospital department with similar clinical characteristics have successfully received OTR4120 treatment. Since this study was completed, over 10,000 patients have been treated with OTR4120 yearly, mostly in the EU and Gulf countries where the device is listed for very hard to heal ulcers, although more and more cases report its efficacy in acute wounds [1] [12]. Its availability and use is progressively extending to other continents, although not yet in the US.

## Conflict of interest

DB is the inventor of the patented RGTA^®^ technology used in this study and has financial interest.
